# Multidisciplinary treatment of patients with rectal cancer: Development during the past decades and plans for the future

**DOI:** 10.3109/03009734.2012.658974

**Published:** 2012-04-19

**Authors:** Bengt Glimelius

**Affiliations:** Department of Radiology, Oncology and Radiation Science, Uppsala University, Akademiska Sjukhuset, Uppsala, Sweden

**Keywords:** Chemoradiotherapy, chemotherapy, local control, multidisciplinary, organ preservation, radiotherapy, randomized trials, rectal cancer

## Abstract

In rectal cancer treatment, both the local primary and the regional and systemic tumour cell deposits must be taken care of in order to improve survival. The three main treatments, surgery, radiotherapy, and chemotherapy, each with their own advantages and limitations, must then be combined to improve results. Several large randomized trials have shown that combinations of the modalities have markedly reduced the loco-regional recurrences, but have not yet had any major influence on overall survival. The best integration of the weakest modality, to date the drugs (conventional cytotoxics and biologicals), is not known. A new generation of trials exploring the best sequence of treatments is required. Furthermore, treatment of rectal cancer is administered to populations of individuals, based upon clinical factors and imaging, and can presently not be further individualized. There is an urgent need to develop response predictors.

## Introduction

Colorectal cancer is the third most common cancer worldwide and the second or third most common cause of cancer death. One-third of the cancers arise in the rectum, the rest in the colon, and virtually all cases are adenocarcinomas. Survival has traditionally been less favourable in rectal than in colon cancer, but this has recently changed. The most likely reasons for the presently slightly better 5-year survival rate in rectal cancer (1–3) are the efforts to decrease rectal cancer local recurrence rates by better staging, improved surgery, and incorporation of radiotherapy. The local recurrence rates have also substantially decreased from 30%–40% a few decades ago down to 10%–15% or even lower in recent studies, and this has had an impact on survival in certain Western populations. Survival has also improved with time for patients treated for colon cancer, but not to the same extent as for rectal cancer (4).

This review about progress in the care of patients with rectal adenocarcinoma is based upon a systematic approach to the scientific literature but gives in addition some personal comments on the development during the past 30 years.

## Diagnosis and staging

Appropriate diagnosis and staging are fundamental as regards choice of therapy. Diagnosis is based on digital rectal examination including rigid sigmoidoscopy with biopsy for histopathological examination. The purpose of the biopsy has so far been to obtain a cancer diagnosis prior to treatment (5). The morphological picture, with the possible exception of poor differentiation, and other cellular or molecular properties of the cancer have had very little impact on treatment decisions. Rectal signet-ring cell carcinomas are considered to have a particularly poor prognosis but may respond well to conventional preoperative therapy (6). Reproducible characteristics with prognostic and/or predictive properties can hopefully soon be identified. At present, the amount of cancer cells in the biopsy should at least be sufficient for an analysis of the KRAS mutational status, since treatment with an epithelial growth factor receptor (EGFR) inhibitor could be an option in the future (7).

Tumours with distal extension to 15 cm or less (as measured by rigid sigmoidoscopy) from the anal margin are classiﬁed as rectal, more proximal tumours as colonic. Whether this 15 cm limit is the best one for choosing a ‘rectal cancer strategy’ or a ‘colon cancer strategy’ can be discussed. Others prefer to separate colon and rectal cancers at the peritoneal reflection, or about 9–12 cm from the anal verge. The localization of the tumour in relation to other organs and structures and, thus, the distance from the anal verge is important for outcome and treatment. From a practical point of view, cancers between 10 and 15 cm are best discussed as rectal cancers since radiotherapy (RT) is an important component of therapy, even if less frequently than for lower rectal cancers (0–10 cm) (8).

Endoscopic ultrasonography for the earliest tumours (cT1-2) or rectal MRI for all tumours is recommended in order to select preoperative treatment and extent of surgery (9,10). The TNM staging system should be used. There is major controversy about which version to use. At present, version 5 from 1997 is preferred by some to the TNM versions 6 (2002) and 7 (2010), since the latter two show marked interobserver variations in defining stage II and stage III (11). At the same time, there is a need for further subclassification, particularly of clinical stage T3 (cT3) in order to individualize therapy, as indicated in Table I.

**Table I.** TNM classification (version 5, 1997) with subclassifications.

TNM	Stage	Extension to	
Tis N0 M0	0	Carcinoma *in situ*: intraepithelial or invasion of lamina propria	
T1 N0 M0	I	Submucosa	
T2 N0 M0	I	Muscularis propria	
T3 N0 M0	IIA	Subserosa/perirectal tissue	
	Substaging^a^	T3a	Less than 1 mm
		T3b	1–5 mm
		T3c	5–15 mm
		T3d	15 + mm
T4 N0 M0	IIB	(b) Perforation into visceral peritoneum; or (a) invasion to other organs^b^	
T1–2 N1 M0	IIIA	1–3 regional nodes involved	
T3–4 N1 M0	IIIB	1–3 regional nodes involved^c^	
T1–4 N2 M0	IIIC	4 or more regional nodes involved	
T1–4 N1–2 M1	IV	Distant metastases	

^a^This subclassification based upon an evaluation using MRI prior to treatment decision is clinically valuable, and used when describing the treatment strategy for primary rectal cancer. It can be used also in the histopathological classification but is not yet validated and therefore not incorporated in any of the TNM versions 5–7.

^b^This is the subclassification in TNM 5. It has been reversed in TNM 6 and 7.

^c^Lymph node classification is modified in TNM 7. Tumour cell deposits without a visible lymph node structure are also considered. It is recommended to investigate at least 12 nodes for proper staging.

## The importance of local control and overall strategy in rectal cancer care

Radical removal of the primary rectal cancer and no local recurrence are prerequisites for cure, although occasional local recurrences can be salvaged by secondary surgery and (chemo)radiotherapy ((C)RT). Avoidance of persistent or recurrent tumour in the pelvis is important, even if cure cannot be achieved, since uncontrolled pelvic growth is usually associated with severe, disabling symptoms. Even if overall survival is not improved, improved local control is a legitimate outcome of different interventions in rectal cancer.

An important aim in rectal cancer is, thus, to treat so that the risk of residual disease in the pelvis is very low or preferably less than about 5% in the population in which curative treatment is intended. This should be possible in all but the few (≤10%) cases who present with a fixed tumour growing into a not readily resectable organ (about half of those with cT4). At the same time, however, as little acute and late morbidity as possible should be aimed at. Surgery, particularly if extensive, may give substantial morbidity, and additional treatments, whether given pre- or postoperatively, increase morbidity. Thus, additional treatments should be chosen with care.

**Figure 1.**
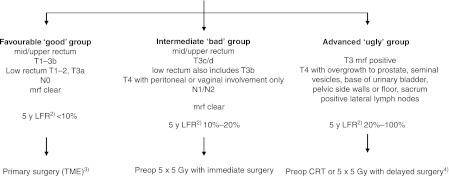
Subgrouping of localized rectal cancer assessed by MRI ^1)^ and recommended primary treatment. ^1)^The algorithm does not primarily address the risk of systemic disease, although this risk also increases with the presence of many of ‘the risk factors’, however, not necessarily parallel to the local failure rate (LFR). The algorithm is also ‘too simplified’ in that also other factors like size of the mesorectum, anterior or posterior location, extramural vascular invasion (EMVI+) are relevant. The recommendations are the ones in use at most centres in Sweden in 2011. ^2)^Calculated in the group of patients planned for surgery, i.e. irrespective of the surgical outcome. The figures are valid if the surgeon is an experienced rectal cancer surgeon and no pretreatment is given. ^3)^A local procedure is possible in a few patients (chiefly pT1, sm1 (+2), N0). This group is in the text referred to as ‘very favourable’. ^4)^CRT means chemoradiotherapy to 50.4 Gy in 1.8 Gy fractions with 5-fluorouracil (capecitabine); 5 × 5 Gy with delayed surgery is used in patients not fit for CRT.

From a practical point of view, rectal cancers can be divided into four groups: very early (some cT1), early (cT1-2, some cT3), intermediate (most cT3, some cT4), and locally advanced (some cT3, most cT4). Factors other than clinical T-stage are also relevant, such as tumour height, closeness to the mesorectal fascia (mrf), potentially the circumferential margin (crm) (preoperatively, the term mrf is better than crm, since the crm cannot be defined until after surgery (12)), nodal (cN)-stage, and vascular and nerve invasion. It is at present not possible to provide a precise description of which T and N substages belong to these groups. The terms ‘favourable or early or good’, ‘intermediate or bad’, and ‘locally advanced or ugly’ can be used for categorizing the rectal cancers into these clinical subgroups. The subdivision presently used in Uppsala and Stockholm, Sweden is shown in Figure 1.

In clinical practice and in many recent studies, the term ‘locally advanced’ has been commonly used for the ‘intermediate/bad’ group but is best reserved for the truly ‘locally advanced/ugly’ tumours (8,13,14). The variability in what is called locally advanced (there is a clear tendency in medicine to use terms that indicate advanced disease even if this is not really present) has been extensive, but there is consensus about the need to subgroup along these lines. Subgrouping is an important step towards individualized medicine. Great discrepancies do, however, exist as regards which treatment is selected for these subgroups between different centres in one country and for different countries.

There is a clear difference in how the regional, subclinical tumour deposits frequently seen in advanced tumours below the peritoneal reflection are managed in Asia and the rest of the world. Should those areas be cleared surgically or by radiation? Surgical removal of the lateral nodes on one or both sides has been the preferred option in Asia, whereas the rest of the world has explored the value of radiation, in addition to surgery for the primary, to kill tumour deposits. Since radiation does not selectively irradiate the lateral nodes, but also includes the primary tumour and the mesorectal nodes, the need for a meticulous surgical dissection technique has not developed as rapidly in the rest of the world as in Asia. Both extensive surgery and additional radiotherapy increase morbidity. The questions are twofold: 1) which of the two alternatives is most efficient in eradicating all tumour cells, i.e. preventing a local failure, and 2) which alternative results in the least morbidity? There are no randomized studies that compare the two strategies. Comparisons between trials reveal that the results are equally good at specialized centres, although patient selection precludes firm conclusions. Theoretically and in clinical practice, it is more efficient to ‘hunt’ subclinical cancer deposits using radiation than using surgery unless you can dissect in a surgical plane. The resultant morbidity is very different, although the impact of this on well-being differs between cultures.

In the Western world where the benefits of radiation therapy to surgery were studied (a preoperative approach was mainly explored in Europe, whereas a postoperative approach was explored in the USA) few small studies indicated that postoperative CRT was better than postoperative RT in preventing local recurrence and that treatment was more effective than no additional treatment. Based upon this evidence, a NIH Consensus Conference and a subsequent NCI report stated that postoperative CRT should be standard treatment in rectal cancer stages II and III (15,16). A small Norwegian trial also found that postoperative CRT was better than no additional treatment, but the evidence for a clear benefit from postoperative CRT considering its toxicity over RT alone has been questioned (17).

In Europe, in contrast, several randomized trials compared surgery alone versus preoperative RT and surgery. These studies showed, particularly if short-course RT (the Swedish 5 × 5 Gy schedule) with immediate surgery was used (18), a relative reduction in local failure rates of 50%–60% in all trials including many hundreds to over a thousand patients. Based upon these experiences, preoperative RT was recommended early on as routine therapy in many countries, but not until quite recently in most countries.

## Pre- or postoperative, short- or long-course, with or without chemotherapy?

For about two decades, four questions have dominated the arena: 1) Should the RT be given before or after surgery? 2) Should it be long-course or short-course? 3) Should the long-course RT be given alone or with chemotherapy? (In Europe researchers were not convinced of the advantages of adding concomitant chemotherapy, as stated in the USdocuments). In addition: 4) As sphincter-saving surgery (SSS) was considered important, could it be increased after preoperative (C)RT? (This was debated extensively and was subject to several trials).

### Pre- or postoperative?

A randomized trial showed at an early date that preoperative short-course RT was more effective than postoperative long-course RT. In that trial (19,20), a brief preoperative schedule was superior to an optimized postoperative high-dose schedule. Subsequently, several trials comparing preoperative CRT with postoperative CRT were initiated. Only one of them completed patient accrual (21). It showed (again) that a preoperative approach was more efficient and less toxic. Superiority of preoperative short-course RT over postoperative CRT was also shown in the MRC-CR07 trial (22). Most of the world has now accepted that additional (C)RT in rectal cancer should be given before rather than after surgery. An analysis of data from all randomized studies also indicated that preoperative RT is more dose-efficient than postoperative RT (23).

### Short- or long-course?

The question of short-course (5 × 5 Gy) versus long-course conventional RT (1.8–2.0 Gy × 25–28) has not yet been settled. A randomized study, the Stockholm III trial, is on-going (700/840 patients have been randomized). In two trials including 316 and 326 patients, respectively, there was no difference in local recurrence rates, disease-free (DFS), and overall survival (OS) between the groups randomized to short-course RT alone or long-course CRT (24,25).

The short-course schedule has gained much popularity in Northern European countries where health care systems are rarely dependent upon private initiatives, whereas the long-course schedule is preferred in countries where physician and hospital budgets are influenced by the number of treatments given. Reimbursement has thus influenced routines, although this has never been officially admitted. Many concerns have been expressed about the long-term consequences of hypofractionated RT. There is considerable evidence that the short-course schedule results in long-term morbidity, and the scale of that morbidity is now well known (26). The long-term morbidity of CRT whether given preoperatively or postoperatively has not been studied systematically, with the result that the extent of late morbidity is not known. Both options, short-course 5 × 5 Gy and long-course CRT are considered valid in the intermediate group of rectal cancers (8).

### Without or with chemotherapy?

The third question, whether or not the long-course RT should be combined with chemotherapy, was answered after the completion of three randomized trials: two in the intermediate group (27,28) and one in the locally advanced, ugly group (29). Local control was better in the combined treatment arm in all three studies, whereas a significant survival gain was only seen in the trial including locally advanced cancers (13,29). Whenever a patient with a locally advanced rectal cancer receives preoperative treatment, CRT should be used unless the patient cannot tolerate this treatment. It should, however, be recognized that the gains from the chemotherapy addition are limited and come with a rather high price.

### Sphincter preservation, organ preservation

Trials, again chiefly run in Europe, have explored whether long-course (C)RT with a delay before surgery could increase SSS rates, whereas others took it for granted that this was the case. Trials have later shown that this effect did not occur to any meaningful extent (30). Hopes about improved chances of SSS influenced routines in many countries, particularly in Southern Europe, Germany, and the USA. At present, hopes about organ preservation (see below) influence treatment decisions in different ways in different parts of the world.

## Need for quality assurance and control

Treatment of rectal cancer is demanding and requires great skill in the entire multidisciplinary team (MDT). Good surgery and good pathology, as well as good radiation techniques and administered chemotherapy, together with long-term complete follow-up, also including functional aspects, are important for quality control. Many countries have recently launched quality assurance (QA) and quality control (QC) programmes in rectal cancer surgery. They have been beneficial for outcome (31,32). Although components other than surgery have been dealt with within the clinical guidelines/care programmes, these, like RT and CRT, must also be fully integrated in the QA and QC programmes.

## Risk-adapted treatment

### Very favourable rectal cancer

In the earliest, most favourable cases, chiefly the malignant polyps (Haggitt 1–3, T1 sm1 (-2?) N0), a local procedure, e.g. using the transanal endoscopic microsurgery (TEM) technique, is appropriate (33,34). The resection should be radical (R0) without signs of vessel invasion or poor differentiation. If this is not the case or if the tumour infiltrates deeper into the submucosa (Haggitt 4, T1 sm(2?-)3) or a T2 tumour, the risk of recurrence because of remaining tumour cells or because of lymph node metastases is too high ( 10%), and the patient should have postoperative CRT or, more safely, be recommended major surgery (total mesorectal excision). If the cancer diagnosis is verified in a biopsy, presurgical CRT is preferred if the intent is to perform a local procedure (33). As an alternative to local surgery, alone or with (preoperative) CRT, local RT (brachytherapy or contact therapy (Papillon technique)) can be used in the most favourable cases. Experience of these treatments is limited outside specialized centres (35), and more prospective studies are required before these techniques can become a part of clinical routines.

### Favourable, ‘good’ rectal cancers

In the early, favourable cases (cT1-2, some early cT3, N0 (cT3a(-b) and clear mrf (mrf-) according to MRI), ‘good’ group) above the levators, surgery alone, meaning a sharp radical dissection using the total mesorectal excision (TME) technique, is appropriate, since the risk of local failure is low (8). Although large randomized trials, where short-course RT has been given, have indicated that this treatment reduces local recurrence rates even further (18,22,36), surgery alone is recommended since the addition of preoperative RT results in overtreatment of too many individuals (8). The balance between the reduction in local recurrence rates and long-term morbidity is intricate.

### Intermediate, ‘bad’ rectal cancers

In the intermediate or ‘bad’ group (most cT3 (cT3(b)c + without threatened or involved mrf (mrf-) according to MRI), some cT4 (e.g. vaginal or peritoneal involvement only), N+), preoperative RT is recommended followed by TME, since this reduces local recurrence rates. Even in the absence of signs of extramural growth on ultrasound or MRI (cT2) in very low tumours (0–5 cm), preoperative RT may be indicated because the distance to the mrf is very small. Treatment with 25 Gy delivered during one week followed by immediate surgery (<10 days from the first radiation fraction) is convenient, simple, and low-toxicity (18,22,36,37). Several trials have shown that the risk of local failure in the randomized population selected for later resection, i.e. the intention-to-treat population, has been reduced by 50%–70% versus surgery alone. More demanding, and not proven more effective, alternatives are 46–50.4 Gy, 1.8–2 Gy/fraction with 5-FU (bolus, continuous infusion, or peroral) (24,25,27,28). Chemotherapy was added to the preoperative radiation primarily based upon extrapolations from the postoperative RT trials in rectal cancer. Two large European trials (27,28) recently showed that the addition of 5-FU improves local control with reduced local failure rates after 5 years. These were 16%–17% in the preoperative RT arms alone and 8%–10% in the CRT arms. In the EORTC trial, the same reduction was seen irrespective of whether the chemotherapy was administered concomitantly or only postoperatively. Two trials have randomized between preoperative 5 × 5 Gy and preoperative CRT (5-FU + 50.4 Gy) without detecting any statistically significant difference in local recurrence rates, DFS, and OS (24,25). In the MRC-CR07 trial including 1350 patients, preoperative 5 × 5 Gy was randomly compared with postoperative CRT if the crm was positive. Local recurrence rates favoured the preoperative arm (5% versus 17%, *P* < 0.001) (22). DFS was also superior in the preoperative arm (hazard ratio (HR) 0.76, *P* = 0.01), whereas OS did not differ significantly (HR 0.91, *P* = 0.04).

### Locally advanced, ‘ugly’ rectal cancers

In the most locally advanced, frequently non-resectable cases (cT3 mrf+, cT4 with overgrowth to other organs (cT4)), preoperative CRT, 50.4 Gy, 1.8 Gy/fraction with concomitant 5-FU-based therapy should be used (8,13,29), followed by radical surgery 6–8 weeks later. In a Nordic randomized trial (cT4NXM0), local control was significantly better after 5 years in the CRT arm (5-FU + 50 Gy) than in the RT only arm (82% versus 67%, *P* = 0.03). Also DFS and cancer-specific survival were significantly better in the combined modality arm, whereas OS did not significantly differ (66% versus 53%, *P* = 0.09) (29).

In very old patients (above 80–85 years) and in patients not fit for CRT, 5 × 5 Gy with a delay of approximately 8 weeks before surgery can be an alternative option, presently under clinical validation after a favourable report first from Uppsala, Sweden and subsequently Leeds, UK (38,39).

### Which chemotherapy schedule? Targeted drugs?

Standard preoperative CRT means a dose of 46–50.4 Gy together with 5-FU given either as bolus injections with leucovorin 6–10 times during the radiation (as in the trials proving that CRT provides better local control than the same RT alone) (13,27-29), prolonged continuous infusion (in all probability better than bolus), or oral capecitabine or UFT. Combinations of 5-FU or other antifolates with other cytostatics, like oxaliplatin or irinotecan, or targeted biological drugs have been extensively explored in phase I–II trials, which have claimed more favourable results (more downsizing, higher pathological complete response (pCR) rates), but also more acute toxicity. Several comparative randomized trials using oxaliplatin are on-going. The initial results of these are not favourable (ACCORD 12/Prodige-2 (40) and SA-01 (41)), and these combinations are still experimental. Nor are the initial results of adding targeted drugs like cetuximab, panitumumab, or bevacizumab favourable (42-46). When cetuximab was added to neoadjuvant oxaliplatin-capecitabine and preoperative CRT in the randomized phase II EXPERT-C trial, more radiological responses were seen in the cetuximab arm (89% versus 72%, *P* = 0.003) than in the KRAS wild-type population (*n* = 90) (47). Overall survival was also improved (96% versus 81% at 3 years, *P* = 0.04).

## Organ preservation?

Apart from the earliest tumours that can be treated with a local procedure or local RT, as described above, it has become increasingly popular to deliver CRT first, wait, and restage the tumour with multiple biopsies/excision biopsy of the previous tumour area (48,49). If no viable tumour cells are then found, no further therapy is delivered (organ preservation), and the patient is monitored closely for at least 5 years. It is then assumed that potential lymph node metastases have been eradicated in parallel with the excellent response of the primary tumour. Although this may undoubtedly occur in some patients, this strategy has not been subject to properly controlled prospective studies. It is likely that this excellent response will not be frequently seen in intermediate and locally advanced cases (50), but rather only in early cases. The advantages (no major surgery and no rectal excision if the tumour is very low) are apparent for certain individuals who run a very high risk from surgery or who cannot accept a stoma. However, the disadvantages for many others are seldom discussed. In most patients with an early ‘good’ rectal cancer, a low anterior resection alone is the preferred therapeutic option. Cure rates are high, and morbidity is only a result of surgery. If these patients are instead treated with the aim of organ preservation, all will receive CRT with its acute morbidity. Those clinically responding very well could then be cared for with a wait-and-see policy. These are the patients who could potentially benefit from this approach, although they would all suffer from the long-term toxicity that can be seen after CRT. This is, as indicated above, not well studied. If the tumour is located in the lower rectum, at least part of the sphincters must be included in the irradiated volume, and poor anal function can be a result. For those not responding well or those recurring during follow-up, major surgery is required. These patients will thus suffer the morbidity inherent to both CRT and surgery. No study has so far had a prospective design so that it is possible to obtain an idea of the proportion of patients who do not require major surgery. With the CRT schedules available today, it is this author's opinion that the group of patients having a true advantage is much smaller than the group of patients who suffers extra morbidity.

### Evaluation of response after preoperative (chemo)radiotherapy

Since the response to preoperative therapy (5 × 5 Gy with a delay or prolonged CRT to 46–50.4 Gy) influences prognosis (51,52) and thus subsequent therapy, both the extent of surgery and postoperative chemotherapy, attempts clinically and pathologically to restage the tumours have been made. There is increasing experience in evaluating tumour response by repeat MRI or PET-CT. Using MRI, a decrease in size can be seen, as well as an increase in fibrosis, and mucous degeneration, indicating response (53). Using FDG-PET, a decrease in uptake can be seen (54-57). At present, the knowledge about the relevance of these changes is too uncertain to modify the extent of surgery. Other attempts to evaluate response after CRT have also been made (58).

Several systems for pathological tumour regression grading have been used (e.g. by Mandard 1994; Dworak 1997; Wheeler 2002; Roedel modification of Dworak 2005). The best approach as regards reproducibility, prognostic information, etc. is not yet known. The tumours should at least be graded into three groups: complete response (pCR), some (potentially in the future, good, moderate, and poor) response, and no response. The proportion of pCRs, meaning absence of tumour cells after a given treatment for a certain substage, is influenced by intensity of dissection. A standardization of the dissection is required if pCR rates are to be used as a valid end-point (59).

## Postoperative adjuvant therapy

Postoperative CRT (e.g. about 50 Gy, 1.8–2.0 Gy/fraction) with concomitant 5-FU-based chemotherapy is, as said above, no longer recommended but could be used in patients with positive crm, perforation in the tumour area, or in other cases with high risk of local recurrence if preoperative RT has not been given. The strategy of giving postoperative CRT to crm + tumours was, however, inferior to giving preoperative 5 × 5 Gy to all, according to the MRC-CR07 trial (22).

Similar to the situation in colon cancer stage III (and ‘high-risk’ stage II), adjuvant chemotherapy can be provided, even if the scientific support for sufficient effect is less than in colon cancer (60,61). In the early chiefly American trials, both chemotherapy and CRT were predominantly given, and thus it was difficult to ascertain which component was responsible for the survival gain (15,16). It is possible that the efficacy of adjuvant chemotherapy is less if the tumour has not responded to the CRT, but this is based only upon a retrospective analysis of one trial (62).

## Radiation therapy volumes and doses

Whenever RT is indicated to lower the risk of local failure in the ‘intermediate/bad’ group or to cause downsizing to allow radical surgery in ‘locally advanced/ugly’ tumours, the primary tumour with the mesorectum and lymph nodes outside the mesorectum, at risk to contain tumour cells, should be irradiated more than exceptionally (63,64). In the ‘early/good’ group before or after a local procedure, only mesorectal nodes are considered at sufficient risk to be involved. The appropriate dose to subclinical disease is not precisely known, but should with 5-FU chemotherapy be at least 46 Gy in 1.8–2 Gy fractions. The relative reduction in local failure rates is then in the order of 50%–60%, and subsequently there is room for improvement. A boost of about 4–6 Gy in 2–4 fractions to the primary tumour is often given, limiting the radiation dose to the entire volume when long-course CRT is given (65).

The entire mesorectum is at great risk of having tumour deposits, often in the mesorectal lymph nodes, in all tumours except the very earliest (T1 sm1 (-2?)) and should be included in the clinical target volume (CTV). An exception is the high tumours where it is sufficient to include the 4–5 cm distal to the tumour. Besides the mesorectal nodes, the presacral nodes along aa. rectales superiores up to the level of S1-2 (if presacral nodes are radiologically involved), the upper border of CTV should be even higher. Local recurrences above S1-2 are rarely seen (66-68). The lateral nodes along aa. rectales inferiores and aa. obturatorii and the internal iliac nodes up to the bifurcation from aa. iliacae communes should be included in tumours below the peritoneal reflection, i.e. in tumours up to about 9–12 cm from the anal verge (69). The risk of lateral node involvement in the Western world is not properly known, but studies from Asia show that these lymph nodes are rarely involved in low–mid rectal pT1-2 tumours and in high tumours irrespective of T-stage (70,71). External iliac nodes should only be included if an anterior organ, like the urinary bladder, prostate, or female sexual organs, is involved to such an extent that there is a risk of involvement of these lymph node stations. The medial inguinal nodes need only to be prophylactically included when the tumour grows below the dentate line (72). When lymph nodes are involved by metastatic disease so that this can be seen on imaging, there is always a risk of aberrant spread, and the CTV can be enlarged to include other nodal stations than those described above.

Fossae ischiorectales should only be included when the levator muscles and the internal and external sphincters are involved since the fascia inside the levators is considered to be a strong barrier to tumour cell penetration (73). Other opinions have been expressed (63).

### Late toxicity from rectal cancer radiotherapy

It is extremely important to know the extent of late toxicity after rectal cancer RT if this is delivered pre- or postoperatively to diminish the risk of local recurrence. The prevention of a local failure must be weighed against the morbidity from (C)RT that all treated patients can develop. Studies have tried to estimate what minimal absolute gain should be present for patients to prefer RT. These studies are very difficult to interpret, although many patients accept an absolute 3% difference for the known morbidity risks of RT (74).

From the Swedish and Dutch randomized trials, we have good evidence of the morbidity after 5 × 5 Gy RT (summarized in (26)). It is beyond the scope of this review to detail this toxicity, but increased risks of poor anal and sexual function, small-bowel toxicity with obstruction, and secondary malignancies have been reported. After having worked with rectal cancer patients for over 30 years, and thus meeting many patients with a local recurrence during the first part of the period, and being actively involved in the research to estimate the extent of late toxicity up to 20 years after the RT, it is my opinion that an absolute risk reduction of approximately 5% motivates the recommendation to irradiate. Furthermore, and very importantly, the RT we give today, and the RT we can routinely give in only a few years, will mean less late toxicity than that seen in the follow-up studies of the RT delivered during the 1980s–1990s (64,75,76).

A very important question as yet unresolved is the late toxicity from 5 × 5 Gy compared with the toxicity seen after 46–50 Gy in 25–28 fractions, usually administered with 5-FU. We know the long-term morbidity from 5 × 5 Gy up to at least 10 years follow-up (with yesterday's techniques) from studies including thousands of patients. We do not have this knowledge from CRT. The Polish (24) and the MRC-CR07 trials (22) have reported late toxicity after 4 years of follow-up, without being able to detect any differences between 5 × 5 Gy and CRT to 46–50 Gy. The short-course schedule uses a high fraction size of 5 Gy, compared with 1.8–2.0 Gy, whereas the total dose is less (25 Gy compared to 46–50 Gy). Both the fraction size and the total dose are relevant. The relationship between total dose, fraction size, and late toxicity is, however, complex.

Another yet unresolved question is whether the addition of 5-FU or in the future other drugs increases late toxicity. In one of the two larger randomized trials in the intermediate risk group (27,28), the addition of 5-FU affected global quality of life (QoL), social functioning, and diarrhoea negatively. Almost 60% of the patients suffered faecal incontinence, impairing their social life (77). In the trial in locally advanced/ugly cancers, more patients had a stoma or a poor anal function in the CRT group than in the RT group (89% versus 70%, *P* = 0.046) (78), but there were no differences in QoL after 4–8 years (79). Whether this means that the chemotherapy addition results in more late toxicity or if this difference reflects survival of patients with more advanced tumours in the CRT group cannot be deduced.

## Conclusions and future development

During the past three decades, I have witnessed the disappearance of a severely disabling condition for many rectal cancer patients, namely a local failure with uncontrolled growth of the cancer in the perineum and pelvis. The surgeon, Åke Rimsten, who in 1978 together with the oncologist Sten Graffman started preoperative radiotherapy in rectal cancer patients in Uppsala, had found that a local rectal cancer failure was the individually most common reason for a patient to occupy a surgical bed in the health care region (unpublished information, see (80)). Together with the surgeon Lars Påhlman, who joined the team in late 1979 as a PhD student, it has been possible during the three decades to improve many details in the total care of these patients. Multiple trials have confirmed the superiority of what we presently consider as recommended care and treatment (Figure 1). A multidisciplinary approach has been "a must" in this development, at present formalized as weekly multidisciplinary team (MDT) meetings, during which all patients are discussed, before the first treatment decision, postoperatively, and at critical time points during the course of the disease.

Practically all details in the care of the patients have been subjected to prospective, frequently randomized trials. With time, you tend to forget selectively, thereby modifying history, but the only research ethics committee approved trial that completely failed was one in patients with a local failure; the patients simply disappeared around 1983–1984. For a variety of reasons, many of the necessary quality improvements, even if proven superior in large randomized phase III trials, have not been introduced at other hospitals until 10–20 years later. It should also be recognized that many uncertainties about what is the best treatment still exist. Furthermore, alternative approaches to attaining low local failure rates, improved survival, together with as few negative consequences from the disease and its treatment as possible, also exist.

The trials have now repeatedly shown that RT, whether alone or with chemotherapy, should be given before surgery to have the best efficacy and least toxicity. This was shown as early as in 1985 but is not until now unanimously agreed upon. It is also my belief that the systemic treatment, being the weakest part of the therapy, should be given before and not after surgery in order to have greatest efficacy. Progression of the local primary tumour should then not occur during the systemic treatment, presently requiring a duration of 5–6 months. The discovery that the short-course schedule resulted in substantial down-staging, was tolerable, and permitted full chemotherapy starting soon after the RT (38), together with the same experiences by other groups, has led to the next generation of studies, such as the ‘Nordic Dream’ or the multicentre ‘RAPIDO’ trial. In the trial where patient inclusion started in June 2011, patients with ‘ugly’ rectal cancers are randomized to the present standard, CRT, surgery and adjuvant chemotherapy (even if not all consider this standard), and an experimental arm with 5 × 5 Gy, neoadjuvant chemotherapy, and surgery at the end.

During the past 30 years, it has also been possible to witness the enormous expansion in knowledge in tumour biology. Better understanding of the molecular mechanisms behind tumour development and progression has created great expectations of improved diagnosis, staging, prognostic evaluation, and selection of the individually best therapy. I have been part of these efforts, attempting to pick up the basic findings and apply or translate them into useful clinical tools. During the course of these efforts, much new and valuable information has been created, but no new clinically valuable markers have been identified. I am not alone in feeling frustrated when the number of mm's from the most peripheral part of the rectal tumour to the mrf (or crm postoperatively) and the ratio of the number of metastatic lymph nodes to the number of sampled nodes are the most informative markers. No predictor of which pre- (or post)operative treatment to choose is available. The efforts to translate basic knowledge into clinically useful information must be intensified or maybe explored along other paths. Sampling of representative and sufficient tumour material not only for diagnosis but also for research prior to, during, and after therapy may help. Functional imaging giving information directly or indirectly on where to sample may also be helpful. Repeated sampling is ethically controversial but could be motivated if coupled to highly skilled research groups both in clinical and preclinical areas. At any rate, we need predictors and must find better ways of identifying them.
